# Supplementary material for: Evolutionary conservation of ubiquitin-like protein urmylation as revealed by URM1 gene shuffle from archaea to yeast
Source: Commun Biol. 2025 Nov 23;8:1637. doi: 10.1038/s42003-025-09212-3 (PMC12645042; doi:10.1038/s42003-025-09212-3)
Supplement: Supplementary file 2 — Supplementary Information [file 42003_2025_9212_MOESM2_ESM.pdf]

Supplementary Information

Evolutionary Conservation of Ubiquitin-like Protein Urmylation as Revealed by *URM1* Gene Shuffle from Archaea to Yeast

Katharina Zupfer[1¶], Lars Kaduhr[1¶], Larissa Bessler[2], Mark Helm[2] and Raffael Schaffrath[1*]

[1] Universität Kassel, Institut für Biologie, FG Mikrobiologie, Heinrich-Plett-Str. 40, 34132 Kassel, Germany

[2] Johannes Gutenberg Universität Mainz, Institut für Pharmazeutische und Biomedizinische Wissenschaften, Staudingerweg 5, 55128 Mainz, Germany

[¶] These authors contributed equally

***** Corresponding author

eMail: schaffrath@uni-kassel.de

Supplementary Tables

Supplementary Table 1. Yeast strains used in this study.

**Yeast strain**	**Genotype**	Reference
BY4741	*MATa his3Δ1 leu2Δ0 met15Δ0 ura3Δ0*	Euroscarf
Y01400	BY4741, *urm1Δ::kanMX4*	Euroscarf
Y01939	BY4741 *uba4Δ::kanMX4*	Euroscarf
FEY15	BY4741, *urm1Δ::kanMX4 uba4Δ::natNT2*	Jüdes et al. (2015)[1]
FEY16	BY4741, *ahp1Δ::kanMX4 urm1Δ::ScHIS3*	Jüdes et al. (2015)[1]
FEY48	BY4741, *urm1Δ::kanMX4 AHP1-(c-myc)$_3$::HIS3MX6*	This study
yKZ2	BY4741, *urm1Δ::kanMX4 tum1Δ::hphMX*	This study
LK45	BY4741, *urm1Δ::kanMX4 uba4Δ::natNT2 ahp1Δ::hphMX*	This study
RK206	BY4741, *urm1Δ::kanMX4 deg1Δ::SpHIS5*	Klassen et al. (2016)[2]

Supplementary Table 2. Plasmids used in this study.

Plasmid	Description	Reference
YEplac195	2µ ori, *ScURA3*	Gietz and Sugino (1988)[3]
pRS313	*ARS1-CEN4*, *ScHIS3*	Sikorski and Hieter (1989)[4]
pRS425	2µ ori, *ScLEU2*	Christianson et al. (1992)[5]
YCplac33	*ARS1-CEN4, ScURA3*	Gietz and Sugino (1988)[3]
YCplac111	*ARS1-CEN4*, *ScLEU2*	Gietz and Sugino (1988)[3]
pAJ16	YCplac111, P_{ADH1}-*UBA4*-T_{CYC1}	Jüdes et al. (2015)[1]
pAJ31	YCplac111, *ScAHP1*	Brachmann et al. (2020)[6]
pAJ33	YCplac111, *ScAHP1-C31S*	Brachmann et al. (2020)[6]
pAJ35	YCplac111, *ScAHP1-K32R*	Brachmann et al. (2020)[6]
pAJ39	YCplac111, *ScAHP1-C62S*	Brachmann et al. (2020)[6]
pAJ45	YCplac33, P_{ADH1}-*3xHA-ScURM1*-T_{CYC1}	Jüdes et al. (2015)[1]
pAJ67	YCplac111, *ScAHP1-C31,62S*	Brachmann et al. (2020)[6]
pKZ2	YCplac33, P_{ADH1}-*3xHA-SaciURM1*-T_{CYC1}	This study
pKZ6	YEplac195, P_{ADH1}-*3xHA-SaciURM1*-T_{CYC1}	This study
pKZ10	YEplac195, P_{ADH1}-*3xHA*-T_{CYC1}	This study
pKZ25	YEplac195, P_{ADH1}-*3xHA-ScURM1*-T_{CYC1}	This study
pKZ48	YEplac195, P_{ADH1}-*3xHA-SaciURM1dG*-T_{CYC1}	This study
pKZ50	YEplac195, P_{ADH1}-*3xHA-ScURM1dG*-T_{CYC1}	This study
pKZ60	pRS313, P_{ADH1}-*3xFLAG-UBA4*-T_{CYC1}	This study
pKZ65	pRS313, P_{ADH1}-*3xFLAG-UBA4-C397S*-T_{CYC1}	This study
pKZ76	YEplac195, P_{ADH1}-*8xHis-TwinStrep-3xHA-ScURM1*-T_{CYC1}	This study
pKZ77	YEplac195, P_{ADH1}-*8xHis-TwinStrep-3xHA-SaciURM1*-T_{CYC1}	This study
pKZ82	YEplac195, P_{ADH1}-*8xHis-TwinStrep-3xHA-ScURM1dG*-T_{CYC1}	This study
pKZ83	YEplac195, P_{ADH1}-*8xHis-TwinStrep-3xHA-SaciURM1dG*-T_{CYC1}	This study
pLK247	YEplac195, P_{ADH1}-*8xHis-TwinStrep*-T_{CYC1}	This study
pQKE	pRS425, *tE(UUC)-tK(UUU)-tQ(UUG)*	Lu et al. (2005)[7]

Supplementary Table 3. Primers used in this study.

Primer	Sequence 5'-3'	Description
SaciUrm1_Fw_ NotI	GGGGCGGCCGCATGAGTGTGAAGATAAGGCT AAAA	Cloning *SaciURM1*
SaciUrm1_Rv_ MlsI	GGGTGGCCATTAGCCACCATGATTTATTGG	Cloning *SaciURM1*
SaciURM1dG_ MlsI_RV	GGGTGGCCATTAACCATGATTTATTGG	Cloning *SaciURM1*dG
ScURM1dG_ MlsI_RV	GGGTGGCCATTTTAACCATGTAATGTTGAAG	Cloning *ScURM1*dG
ScUrm1_NotI_ Fw	GGGGCGGCCGCATGGTAAACGTGAAAGTGGA G	Cloning *ScURM1*
ScUrm1_MlsI_ Rv	GGGTGGCCATTTTAACCACCATGTAATGTTG	Cloning *ScURM1*
UBA4_C397S_ FW	CAGTAATATAGTGATTCTTTCCCGCTACGGTAA CGACTCTC	SDM Uba4 C397S
UBA4_C397S_ RV	GAGAGTCGTTACCGTAGCGGGAAAGAATCACT ATATTACTG	SDM Uba4 C397S
pTH1_pADH_ BamHI_Fw	GGGGGGATCCCAACTTCTTTTCTTTTTTTTTCT	Cloning Uba4
UBA4_EcoRI_ RV	GCTGAATTCTTACTAATATTTAGGAATGGTTTGA TCAATATCGTC	Cloning Uba4
AHP1KOF	ATTTCAACAAACCAGAACAACACAAGTACTACC AATAACCACAACAAAACCAGCTGAAGCTTCGTA CGC	*AHP1* ko
AHP1KOR	TTTTGAATTTTTTTTATATAAACATGGTTTTATTG TCTATTACATAGCATGCATAGGCCACTAGTGGA TCTG	*AHP1* ko
AHP1-(c-myc)9_FW	CCTCTCCCTTGCCAATTGTG	*AHP1* ve
AHP1_RV_ EcoRI	GGGGAATTCCTGCTCCAACTCACTCTGTC	*AHP1* ve
KO_TUM1_FW	ACAATGAGGACAAAAGCATAAAGTTGTGAAGAA AATTGCCCATACATTCACAGCTGAAGCTTCGTA CGC	*TUM1* ko
KO_TUM1_RV	TTAATATATGTAGCTAAATAAATCGACTTGTCAA GAATATATTTCTCTTAGCATAGGCCACTAGTGG ATCTG	*TUM1* ko
N_TUM1_FW	GTCGTGGTAAGTCCTGTTCC	*tum1* ve
N_TUM1_RV	TCCCTCGATAGAAAGGTCTC	*tum1* ve
UBA4_FC_Fw	CATACAATCAACTCTCCATGATGAATGACTACC ATCTCGAG	FC Uba4

UBA4_FC_Rv	CCACTAGCAGCAACTCTCGAATATTTAGGAATG GTTTGATCAATATCGTC	FC Uba4
N-terminal FLAG FW	TACCAAGCATACAATCAACTATCTCATATCATAT GGACTACAAAGACCATGACGGTGATTATAAAGA TCATGACATCGATTACAAGGATGACGATGACAA GGGTTCTTCTGGTAATGACTACCATCTCGAGGA TACCACG	N-terminal FLAG tagging
N-terminal FLAG RV	CGTGGTATCCTCGAGATGGTAGTCATTACCAG AAGAACCCTTGTCATCGTCATCCTTGTAATCGA TGTCATGATCTTTATAATCACCGTCATGGTCTTT GTAGTCCATATGATATGAGATAGTTGATTGTAT GCTTGGTA	N-terminal FLAG tagging
M13_FW_-40	GGTTTTCCCAGTCACGAC	pc
M13_RV_-27	GGAAACAGCTATGACCATG	pc
ADH C-term FW	GCTATCAAGTATAAATAGACCTGCA	Sequencing
N_Uba4/MOCS 3 RV	TACATGCGTACACGCGTCTG	Sequencing

Abbreviations: FastCloning (FC), knock-out (ko), ko verification (ve), site-directed mutagenesis (SDM), plasmid construction (pc)

Supplementary Table 4. Individual data points of LC-MS/MS analysis of tRNA wobble uridine (U_{34}) modifications.

Strain	***urm1*∆ + *ev***		
U_{34} modification	**ncm^5U_{34}**	**mcm^5U_{34}**	**$mcm^5s^2U_{34}$**
% mod / U n = 3	0,3428	1,0619	n.d.
	0,2923	0,9136	n.d.
	0,2890	0,8841	n.d.
Mean	0,3080	0,9532	n.d.
± SD	0,030194727	0,095321582	n.d.
Strain	***urm1*∆ + *ScURM1***		
U_{34} modification	**ncm^5U_{34}**	**mcm^5U_{34}**	**$mcm^5s^2U_{34}$**
% mod / U n = 3	0,3045	0,5329	0,3720
	0,2750	0,4321	0,3693
	0,2674	0,4671	0,3010
Mean	0,2823	0,4774	0,3474
± SD	0,019587686	0,051189278	0,040216292
Strain	***urm1*∆ + *SaciURM1***		
U_{34} modification	**ncm^5U_{34}**	**mcm^5U_{34}**	**$mcm^5s^2U_{34}$**
% mod / U n = 3	0,3824	1,1800	n.d.
	0,3088	0,9963	n.d.
	0,3066	0,9061	n.d.
Mean	0,3326	1,0275	n.d.
± SD	0,04313608	0,13959142	n.d.

Supplementary Figures

Supplementary Figure 1

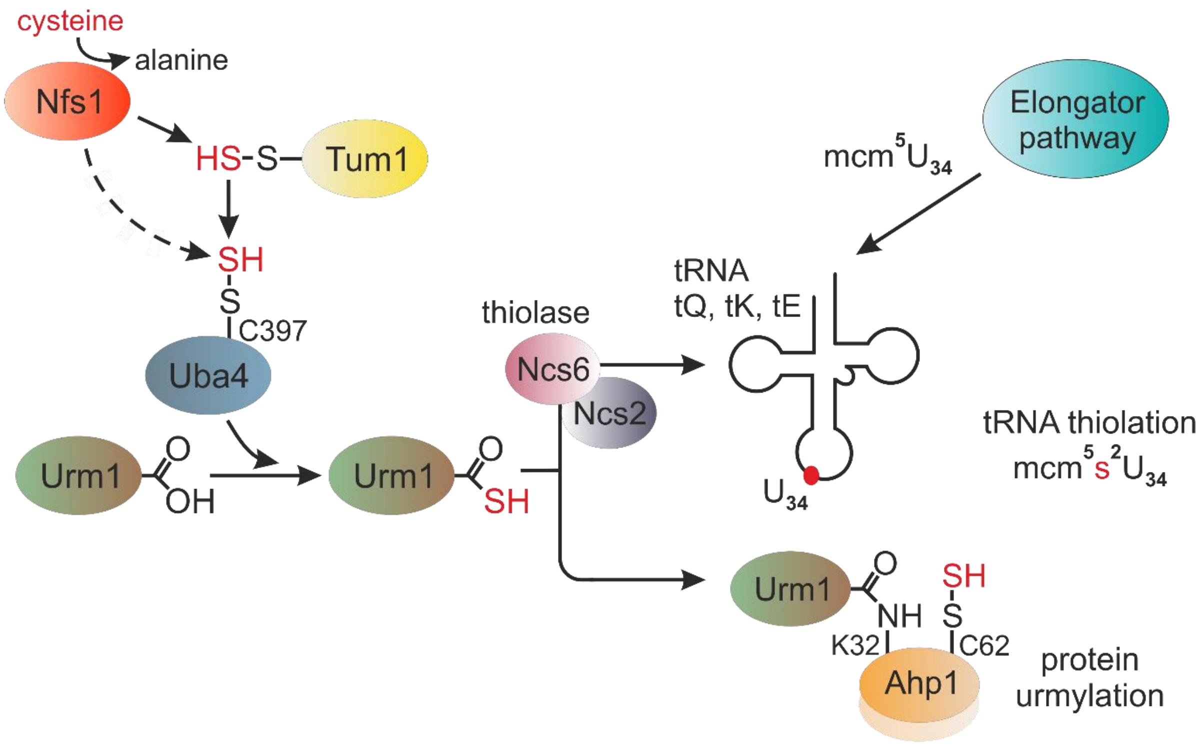

Supplementary Figure 1. Scheme of the yeast Urm1 pathway. Sulfur flow (red) required for Urm1 activation starts with sulfur extraction from cysteine through desulfurase Nfs1, followed by direct (Nfs1) or indirect (Tum1) sulfur transfer to the catalytic active cysteine (C397) of E1-like enzyme Uba4 that thiocarboxylates the C-terminus of Urm1. Sulfur-activated Urm1 provides sulfur for thiolase complex (Ncs6/Ncs2) resulting in s^2U_{34} modification on tRNAs (tQ, tK, tE), in conjunction with the Elongator pathway and methyltransferase complex Trm9-Trm112 (not shown), resulting in $mcm^5s^2U_{34}$ modification. Thiocarboxylated Urm1 can also transfer sulfur for Ahp1 cysteine persulfidation on C62 followed by lysine-directed Ahp1 urmylation on K32. Scheme modified according to Jüdes *et al.* (2016)[8] and Ravichandran *et al.* (2022)[9].

Supplementary Figure 2

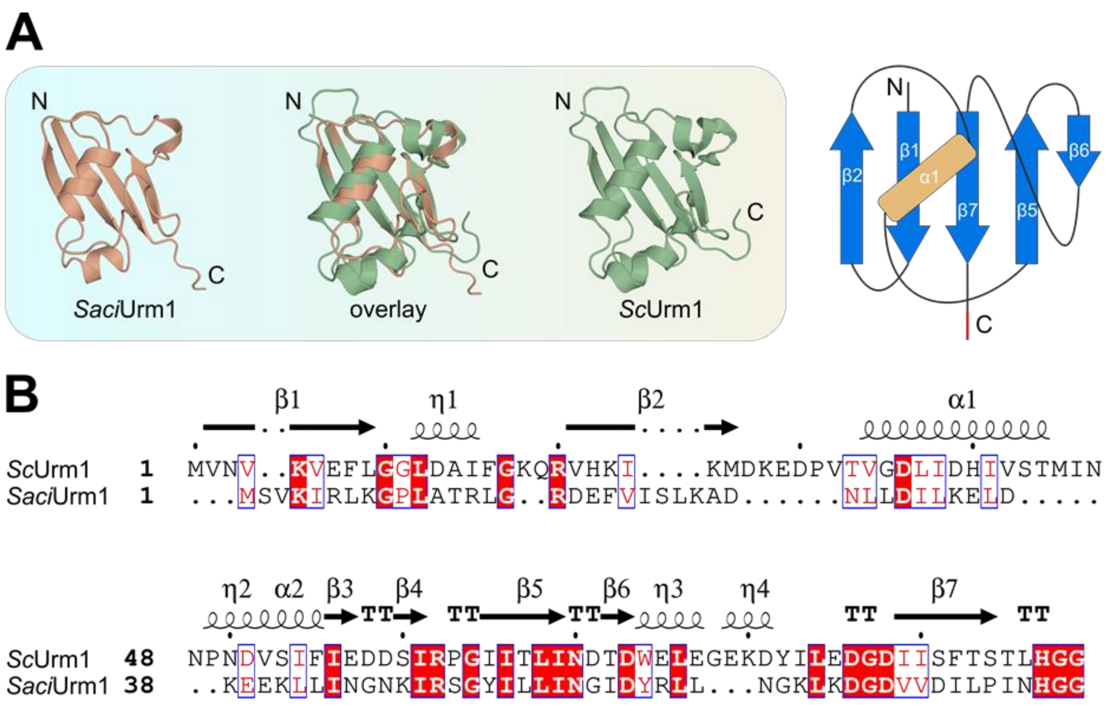

Supplementary Figure 2. Structure and sequence comparison of Urm1 from *S. cerevisiae* (*Sc*) and *S. acidocaldarius* (*Saci*). (**A**) Structures of *Saci*Urm1 (Saci_0669) in light brown modeled with AlphaFold[10] and *Sc*Urm1 (PDB: 2qil) in green are displayed individually and as overlay. A scheme of the highly conserved β-grasp fold with five antiparallel β-sheets (marine) and central α-helix (lightorange) is shown on the right. (**B**) Sequence alignment of *Sc*Urm1 and *Saci*Urm1. Red boxes with white characters indicate strict identity, while red characters show similarity in a group, and blue frames display similarities across groups. Predicted secondary structures are α-helices (α), β-sheets (β), 310-helices (η) and strict β-turns (TT).

Supplementary Figure 3

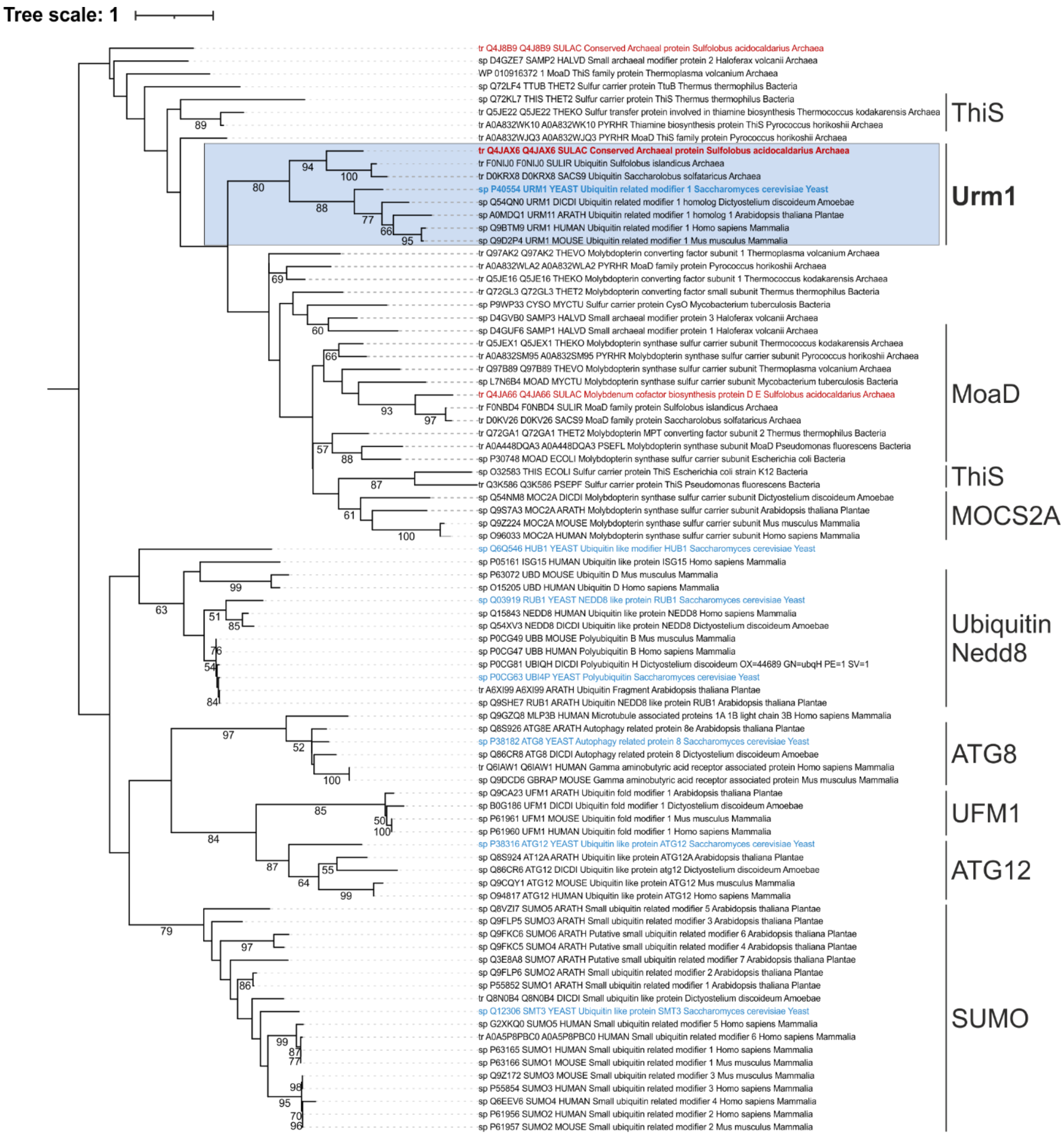

Supplementary Figure 3. Phylogenetic tree of ubiquitin-like proteins from Eukarya, Archaea and Bacteria. Multiple sequence alignment was trimmed (Noisy v1.5.12.1[11,12]) and used in maximum likelihood analyses (RAxML v8.2.12[13] implemented in raxmlGUI2.0.13[14]). Numbers on nodes indicate bootstrap support values >50%. The monophyletic Urm1 group is highlighted in a blue box. Analyzed protein sequences of *S. acidocaldarius* are highlighted in red and *S. cerevisiae* sequences in blue.

Supplementary Figure 4

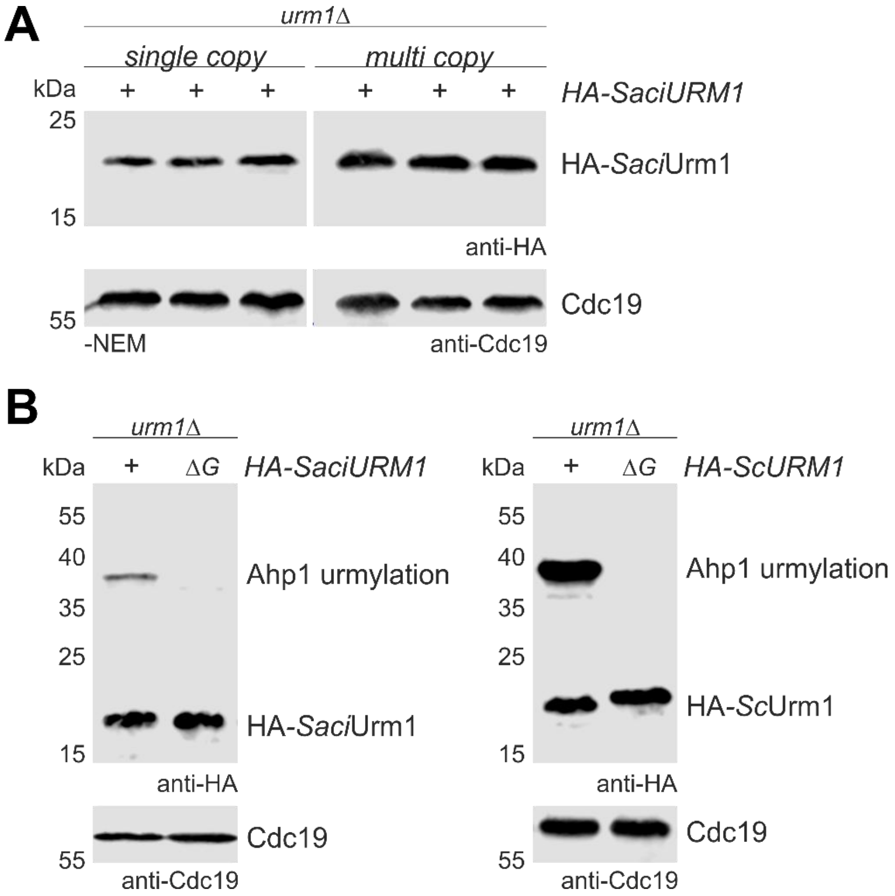

Supplementary Figure 4. Expression of *Saci*Urm1 in yeast and C-terminal glycine dependent Ahp1 urmylation. (**A**) Expression of *Saci*Urm1 in yeast. Western blot under non-conjugating (-NEM) conditions with protein extracts from biological *urm1Δ* cell triplicates expressing *HA-SaciURM1* from single-copy (left) or multi-copy (right) plasmids. *Saci*Urm1 (~20 kDa) was detected using anti-HA Western blots (top panel). The anti-Cdc19 blots (bottom panel) were used to control protein loading. (**B**) C-terminal glycine residues in *Saci*Urm1 and *Sc*Urm1 are essential for urmylation. Western blots under reducing and conjugating conditions with protein extracts from *urm1Δ* cells expressing *HA-SaciURM1* (left) or *HA-ScURM1* (right) and their respective C-terminal glycine deletion mutants (ΔG). The anti-HA antibody detects free *Saci*Urm1 (~20 kDa) and *Sc*Urm1 (~23 kDa) as well as their truncated versions and urmylated Ahp1 (~40 kDa) (top panels). Anti-Cdc19 served as loading control (bottom panels).

Supplementary References

1. Jüdes, A. *et al.* Urmylation and tRNA thiolation functions of ubiquitin-like Uba4·Urm1 systems are conserved from yeast to man. *FEBS Lett* **589**, 904–909 (2015).
2. Klassen, R. *et al.* tRNA anticodon loop modifications ensure protein homeostasis and cell morphogenesis in yeast. *Nucleic Acids Res* **44**, 10946–10959 (2016).
3. Gietz, R. D. & Sugino, A. New yeast-Escherichia coli shuttle vectors constructed with in vitro mutagenized yeast genes lacking six-base pair restriction sites. *Gene* **74**, 527–534 (1988).
4. Sikorski, R. S. & Hieter, P. A system of shuttle vectors and yeast host strains designed for efficient manipulation of DNA in Saccharomyces cerevisiae. *Genetics* **122**, 19–27 (1989).
5. Christianson, T. W., Sikorski, R. S., Dante, M., Shero, J. H. & Hieter, P. Multifunctional yeast high-copy-number shuttle vectors. *Gene* **110**, 119–122 (1992).
6. Brachmann, C. *et al.* Redox requirements for ubiquitin-like urmylation of Ahp1, a 2-Cys peroxiredoxin from yeast. *Redox Biol* **30**, 101438 (2020).
7. LU, J., HUANG, B., ESBERG, A., JOHANSSON, M. J. O. & BYSTRÖM, A. S. The Kluyveromyces lactis γ-toxin targets tRNA anticodons. *RNA* **11**, 1648–1654 (2005).
8. Jüdes, A., Bruch, A., Klassen, R., Helm, M. & Schaffrath, R. Sulfur transfer and activation by ubiquitin-like modifier system Uba4•Urm1 link protein urmylation and tRNA thiolation in yeast. *Microb Cell* **3**, 554–564 (2016).
9. Ravichandran, K. E. *et al.* E2/E3-independent ubiquitin-like protein conjugation by Urm1 is directly coupled to cysteine persulfidation. *EMBO J* **41**, e111318 (2022).
10. Jumper, J. *et al.* Highly accurate protein structure prediction with AlphaFold. *Nature* **596**, 583–589 (2021).
11. Dress, A. W. M. *et al.* Noisy: identification of problematic columns in multiple sequence alignments. *Algorithms Mol Biol* **3**, 7 (2008).
12. Grünewald, S., Forslund, K., Dress, A. & Moulton, V. QNet: an agglomerative method for the construction of phylogenetic networks from weighted quartets. *Mol Biol Evol* **24**, 532–538 (2007).
13. Stamatakis, A. RAxML version 8: a tool for phylogenetic analysis and post-analysis of large phylogenies. *Bioinformatics* **30**, 1312–1313 (2014).
14. Edler, D., Klein, J., Antonelli, A. & Silvestro, D. raxmlGUI 2.0: A graphical interface and toolkit for phylogenetic analyses using RAxML. *Methods in Ecology and Evolution* **12**, 373–377 (2021).

Supplementary Raw Data

Raw data for Figure 2A:

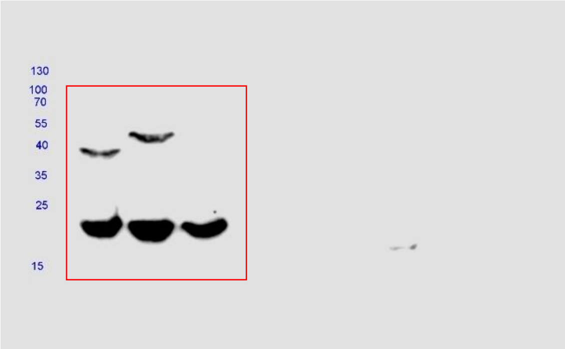

Blot for Figure 2A: anti-HA

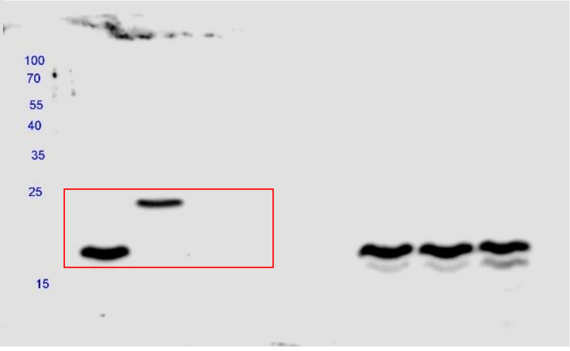

Blot for Figure 2A: anti-Ahp1

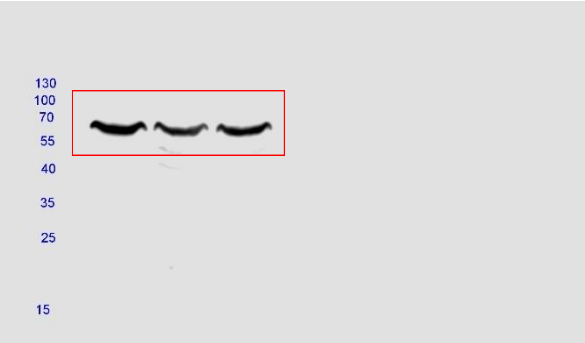

Blot for Figure 2A: anti-Cdc19

Raw data for Figure 2B:

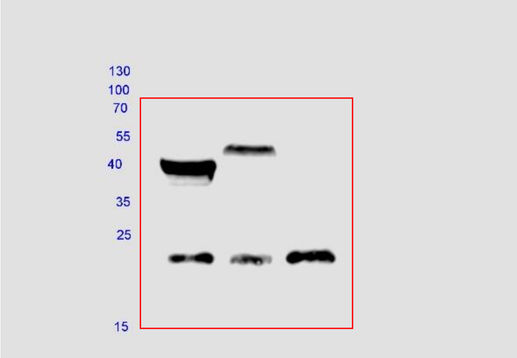

Blot for Figure 2B: anti-HA

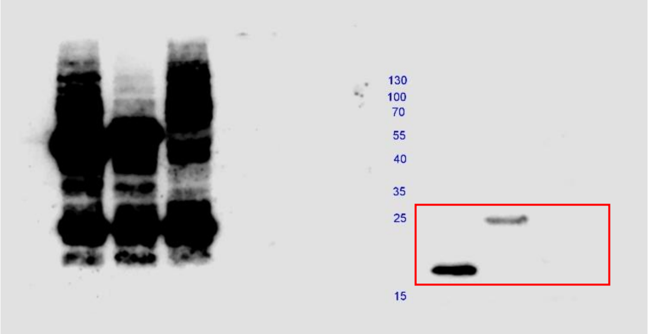

Blot for Figure 2B: anti-Aph1

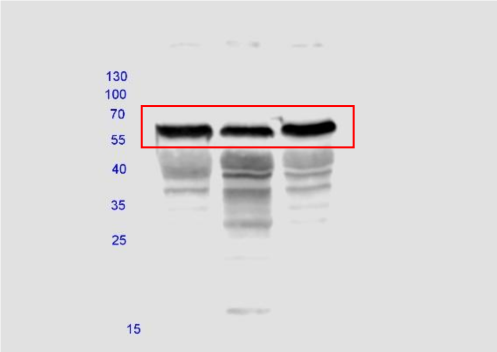

Blot for Figure 2B: anti-Cdc19

Raw data for Figure 2C:

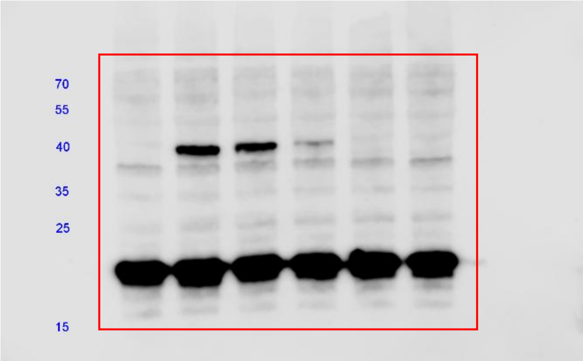

Blot for Figure 2C: anti-HA

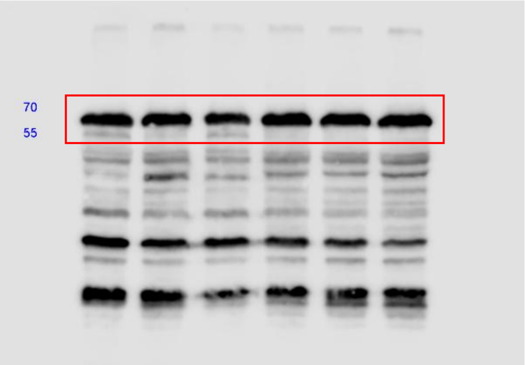

Blot for Figure 2C: anti-Cdc19

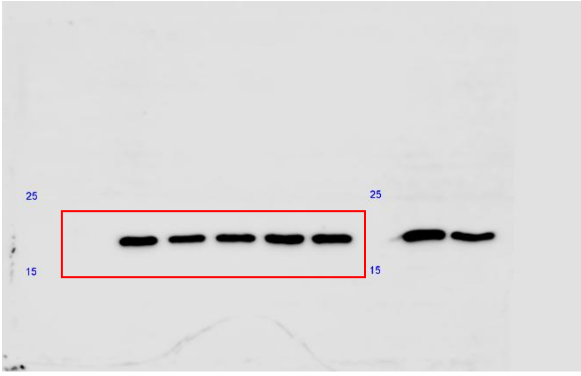

Blot for Figure 2C: anti-Ahp1

Raw data for Figure 3B:

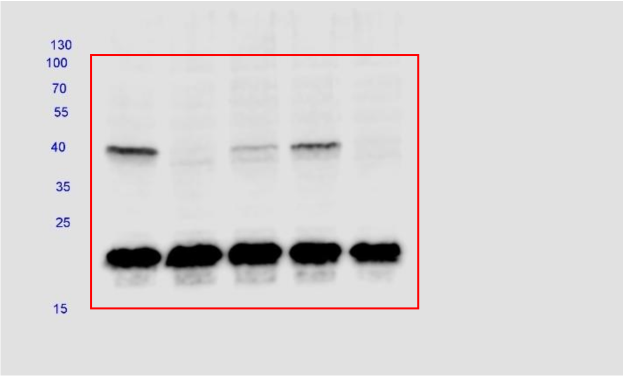

Blot for Figure 3B: anti-HA

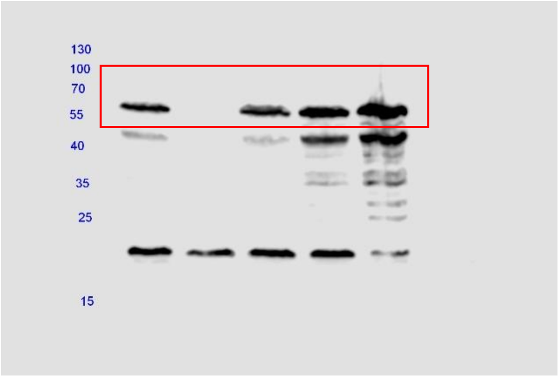

Blot for Figure 3B: anti-FLAG

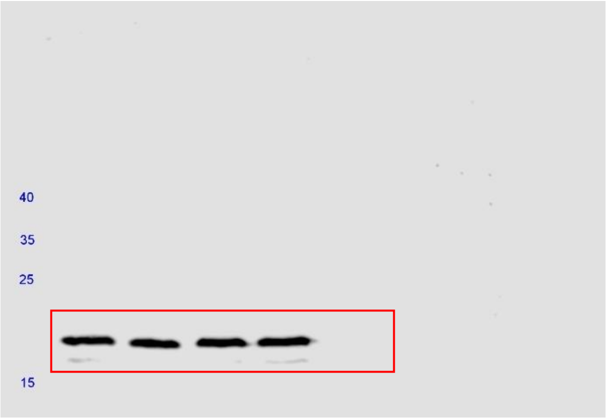

Blot for Figure 3B: anti-Ahp1

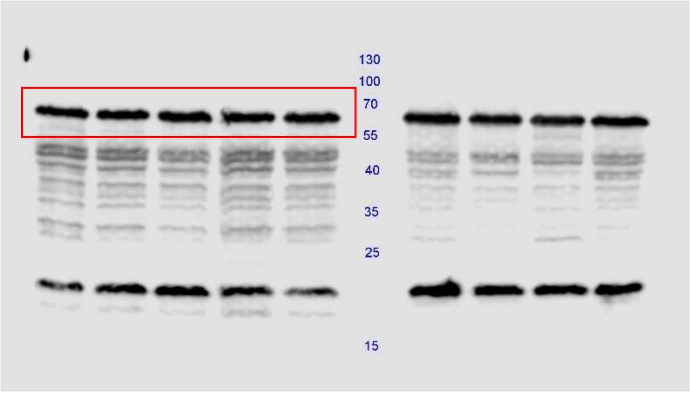

Blot for Figure 3B: anti-Cdc19

Raw data for Figure 4B:

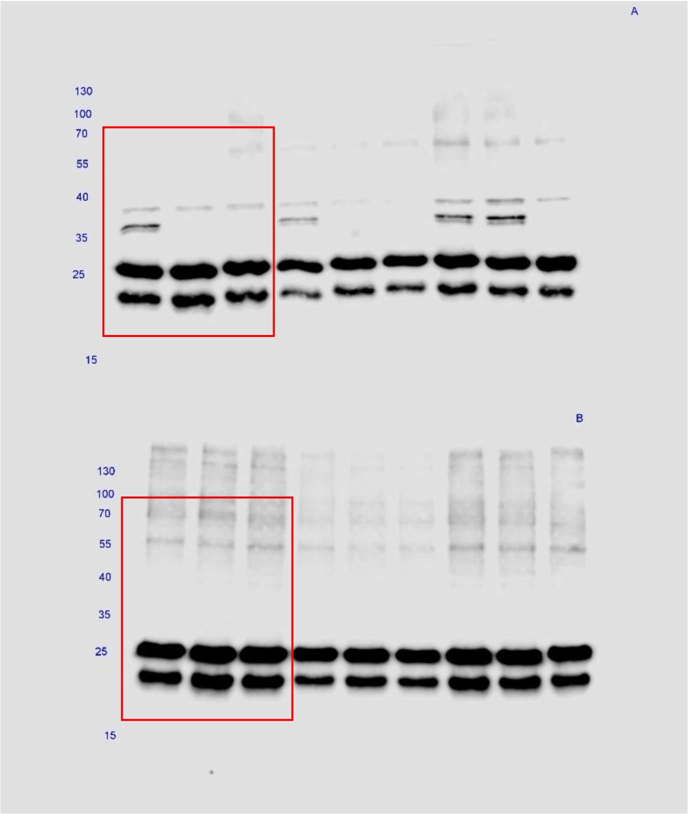

Blot for Figure 4B: anti-HA

Raw data for Figure 4C:

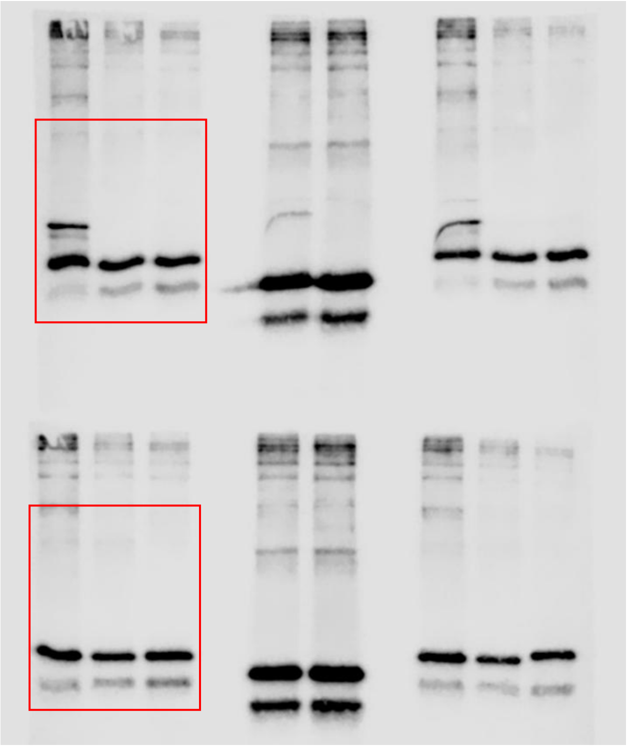

Blot for Figure 4C: anti-HA

Raw data for Figure 5B:

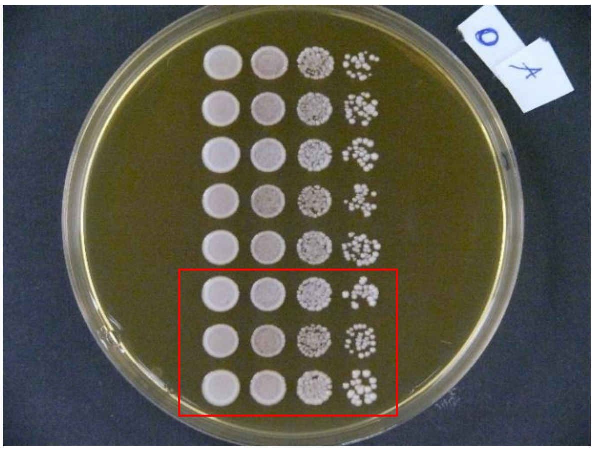

Phenotypical growth test Figure 5B: control

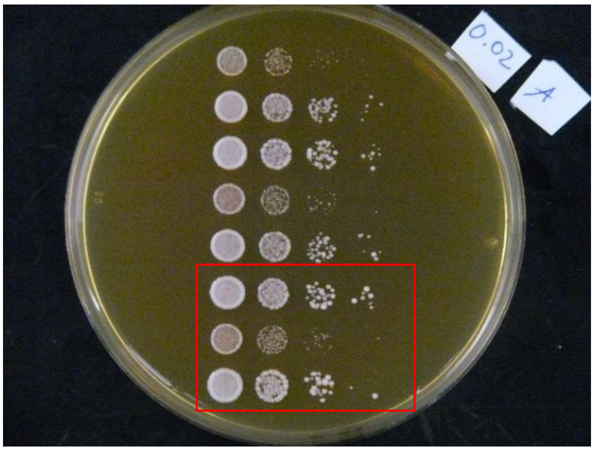

Phenotypical growth test Figure 5B: +zeocin

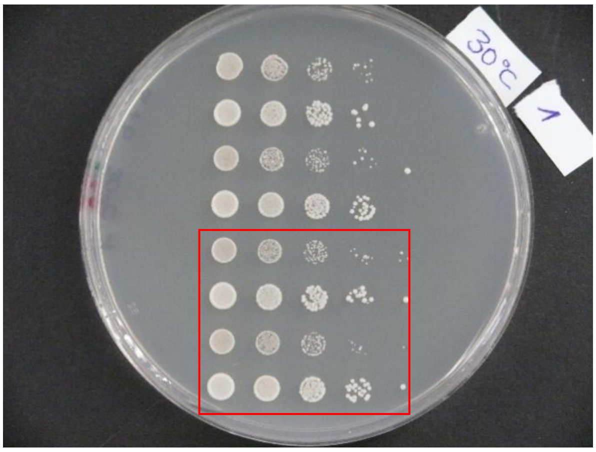

Phenotypical growth test Figure 5B: 30°C

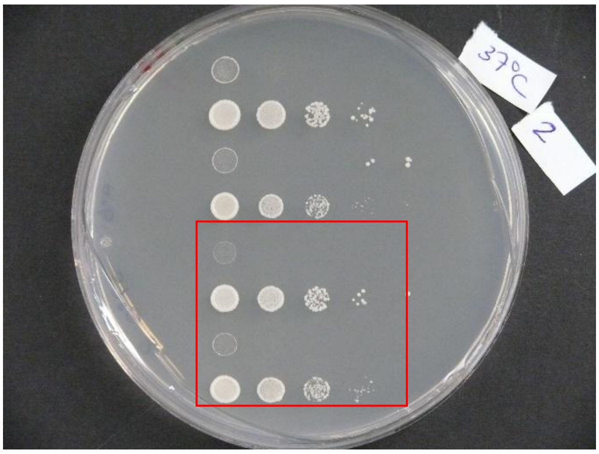

Phenotypical growth test Figure 5B: 37°C

Raw data for Supplementary Figure 4A:

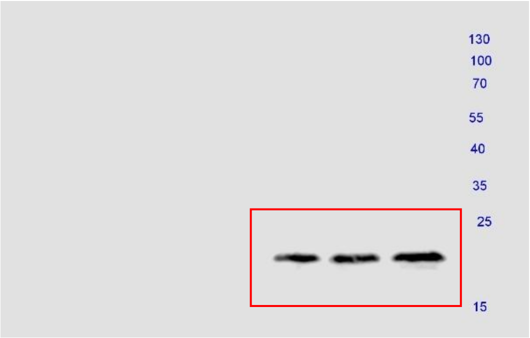

Blot for Figure S4A: anti-HA

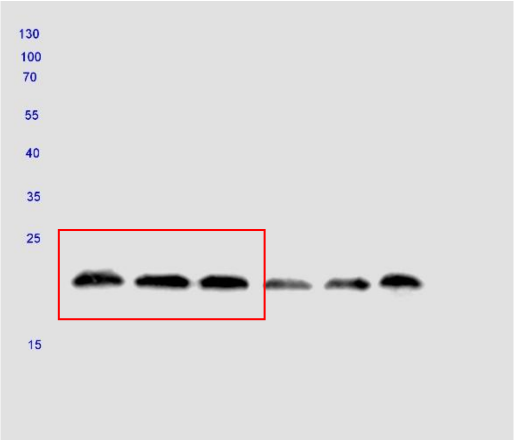

Blot for Figure S4A: anti-HA

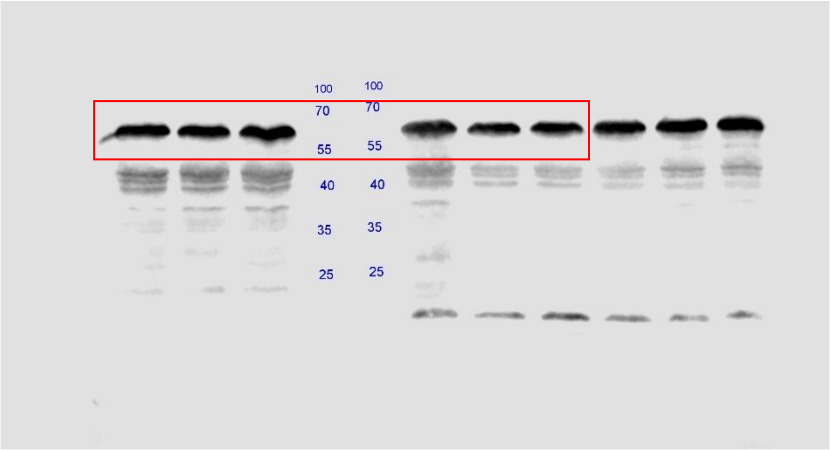

Blot for Figure S4A: anti-Cdc19

Raw data for Supplementary Figure 4B:

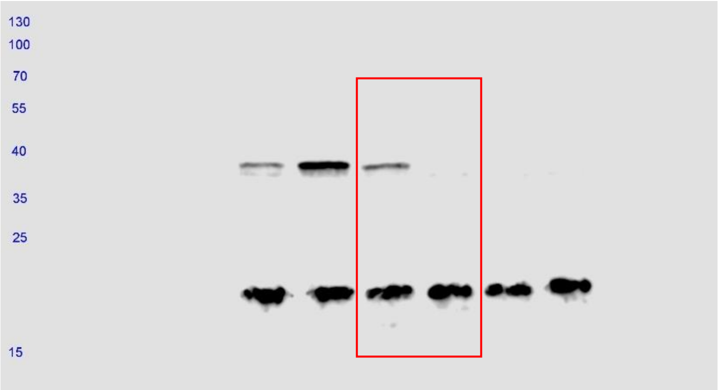

Blot for Figure S4B: anti-HA

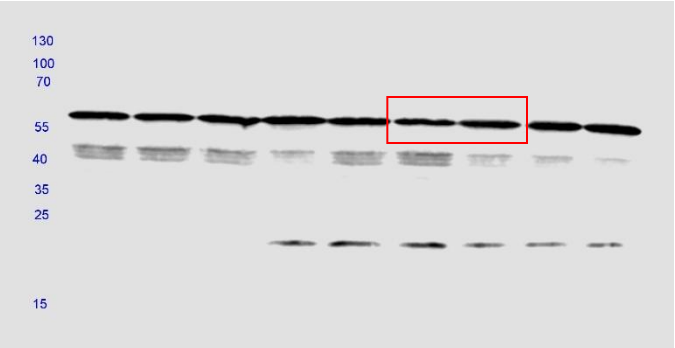

Blot for Figure S4B: anti-Cdc19

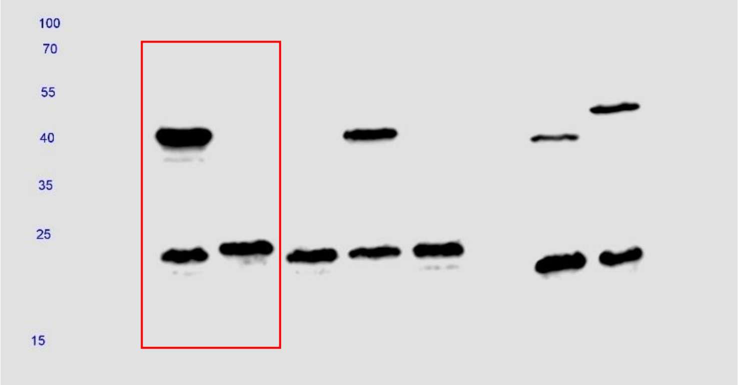

Blot for Figure S4B: anti-HA

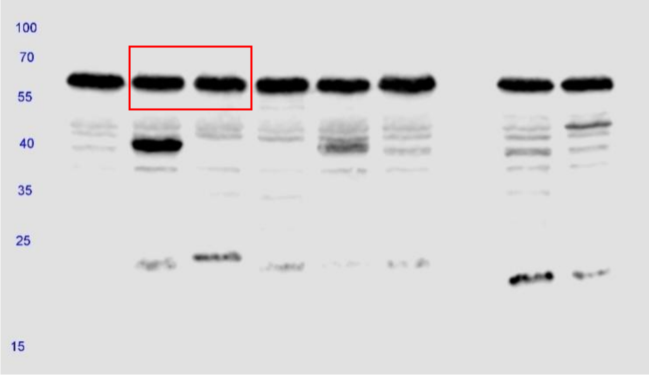

Blot for Figure S4B: anti-Cdc19
