# Supplementary material for: Evolutionary conservation of ubiquitin-like protein urmylation as revealed by URM1 gene shuffle from archaea to yeast
Source: Commun Biol. 2025 Nov 23;8:1637. doi: 10.1038/s42003-025-09212-3 (PMC12645042; doi:10.1038/s42003-025-09212-3)
Supplement: Supplementary file 3 — Description of Additional Supplementary Files [file 42003_2025_9212_MOESM3_ESM.pdf]

Description of Additional Supplementary files

File name: Supplementary Data

Description: Source data behind the graph in Figure 5A in the paper.
